# High school and transition experiences of twice exceptional students with autism spectrum disorder: Parents’ perceptions

**DOI:** 10.3389/fpsyg.2022.995356

**Published:** 2022-09-26

**Authors:** Joseph Madaus, Emily Tarconish, Shannon W. Langdon, Nicholas Gelbar

**Affiliations:** ^1^Department of Educational Psychology, University of Connecticut, Storrs, CT, United States; ^2^Department of Special Education, University of Illinois at Urbana-Champaign, Champaign, IL, United States

**Keywords:** ASD, twice exceptional, secondary transition, parent perceptions, high school

## Abstract

Students with autism spectrum disorder (ASD) are accessing college in increasing numbers. Within this cohort are students who are twice exceptional—those who are both academically talented and diagnosed with ASD. Little is known about factors and experiences that impact their successful transition to college. Parents play a critical role in the secondary transition process, but currently, there is a paucity of research that examines their perceptions of this experience. This study presents the results of semi-structured interviews with the parents of 10 college students with ASD who were enrolled in postsecondary institutions in the United States. The parents provided perceptions of their student’s best and most challenging aspects of high school, as well as of the transition preparation the student received. Suggestions were offered in regard to how transition services can be improved for this group of students. Implications for planning and practice are presented.

## Introduction

Autism spectrum disorders (ASDs) are a complex set of neurodevelopmental conditions that are characterized by a variety of strengths and challenges (Pena and Kocur, 2013). ASD is commonly described through communication, social, and behavioral difficulties. These impediments can be demonstrated in changes in routine, emotional regulation, engagement in repetitive behaviors, and restricted interests, as well as the development of relationships with others (U.S. Department of Health and Human Services, 2020). Some students with ASD^1^ have reported that they struggle maintaining friendships, while others have reported that having a positive peer relationship or a friend in school made going to school more enjoyable and improved their overall wellbeing (Cridland et al., 2015; Saggers, 2015; Bolic Baric et al., 2016; Goodall and MacKenzie, 2019). Students with ASD also may have difficulties learning, which may impact their academic achievement. Individuals with ASD have reported having difficulty maintaining concentration in the classroom, may fail to complete assignments, and struggle in specific subjects (e.g., math and literacy-based courses; Bolic Baric et al., 2016). Difficulties with academics often increase with age as they are related to the pace of the work, the workload, pace, independence, and pressure of grades (Saggers, 2015; Bolic Baric et al., 2016).

Individuals with ASD also have a range of strengths and can be highly gifted individuals (Reis et al., 2014). For example, individuals with ASD can develop proficiencies and talents relating to areas of special interests and can creatively focus on these in great detail (Asperger/Autism Network, 2016). Academically, individuals with ASD often become proficient on one specific domain in which they have more specified abilities (Meilleur et al., 2015). These areas of strength make postsecondary education a desirable option for some students with ASD, and approximately 57.4% of students with ASD exiting high school are focused on postsecondary education (Wei et al., 2015).

## Twice exceptionality

In the K-12 education system in the United States, twice exceptional (2e) students are often described as those who demonstrate the potential for high achievement or creative productivity in one or more domains such as math, science, technology, and visual, spatial, or performing arts as well as manifesting one or more disabilities defined by federal eligibility criteria under the Individuals with Disabilities Education Act (2004) (IDEA) or Section 504 of the Rehabilitation Act of 1973 (Reis et al., 2014; Dare and Nowicki, 2015). Specific disabilities may include ASD, specific learning disabilities, speech and language disorders, emotional/behavioral disorders, physical disabilities, or other health impairments (Reis et al., 2014). Students who are twice exceptional are often difficult to diagnose as their high abilities may mask their disability or their disability may mask their high abilities (Assouline et al., 2012; Reis et al., 2014; Dare and Nowicki, 2015). Masking is a phenomenon in which cognitive strengths compensate for weaknesses, or weaknesses overshadow cognitive strengths (Bechard, 2019). Moreover, students who are twice exceptional may struggle to demonstrate their abilities.

### Twice exceptional students with autism spectrum disorder

Currently, we understand little about the experiences, strengths, and challenges faced by academically talented students with ASD (hereafter referred to as 2e-ASD) who are transitioning to college. There is limited literature in this area, and there are no firm data on prevalence as no federal agencies gather base rate data for this group of students (National Association for Gifted Children, 2009).

Students with 2e-ASD face several challenges academically and socially. One common challenge is the lack of identification for either giftedness or disability as many school districts do not appropriately support students who are both gifted and have a disability (Neumeister et al., 2013; Bechard, 2019), and lack of identification can lead to lack of access to services and supports. Challenges for those who are not appropriately identified may include difficulty communicating with others, social skills, sensory integration, behaviors, generalization, working memory, processing speed, and understanding the concept of time (Dare and Nowicki, 2015; Rubenstein et al., 2015), which can lead to negative outcomes such as feelings of academic ineptitude, anxiety, or fear of failure in academic tasks, and academic achievement (Dare and Nowicki, 2015). Specifically, students often face high levels of anxiety, poor self-concepts, and deficits in executive functioning because of the inconsistencies in their academic performance (Neumeister et al., 2013; Reis et al., 2014). These difficulties can impact the students’ ability to socialize due to inability to find intellectual peers or if the students hyperfocus on a topic of interest or appear inflexible to others if they feel their idea is perfect (Reis et al., 2014).

Individuals’ ability to hyperfocus can be considered a strength. This ability allows students to delve into specific topics and become experts in a particular area. Students’ talents and gifts should be supported within their academics and interactions with peers, and students with 2e-ASD benefit from being allowed to focus on their specific talents and interests (Rubenstein et al., 2013; Reis et al., 2014). These students often require accommodations and/or modifications to their instruction and curriculum including specialized instruction, accelerated learning opportunities, direct services, and opportunities to develop their talents but also meet their other needs (Reis et al., 2014). However, Individualized Education Programs and Section 504 accommodation plans are written to address the disability and not to develop the students’ talents (Reis et al., 2014).

However, research is also clear that effective practices with these students should balance developing students’ academic strengths and interests with learning to work with the area(s) of difficulty (Baum et al., 2014). It has been recommended that for accommodation/modification plans to be successful for these students, more attention should be focused on developing talents while also attending to the disability (Reis et al., 2014). In regard to 2e-ASD secondary students who seek to attend postsecondary education, strength-based transition planning, an evidence-based practice, enables students to increase their self-determination skills and to identify their postsecondary goals based on their individual strengths, interests, and preferences (Hatfield et al., 2018). When preparing students for college, it is imperative for students with ASD to develop self-efficacy and self-disclosure skills, as roughly only 62.7% of students with ASD who attend postsecondary education report their disability and receive services (Newman et al., 2011).

## Parents’ perspectives

Parents have a substantial influence on their child’s education as they often choose the school their child attends, provide access to out-of-school resources, and advocate for in-school supports (Neumeister et al., 2013; Rubenstein et al., 2015). Importantly, per the regulations of IDEA in the United States, parents are also specifically part of the student’s special education team in the K-12 special education and are deeply involved in the secondary transition process. Parents can serve as strong advocates for their children and may spend time educating professionals who work with their child (Morrison et al., 2009; Rubenstein et al., 2015). This influence is likely to continue when their child enters postsecondary education; however, their involvement may look different post-high school. Many parents have reported feeling that they were “fighting the system” at all prior levels of school and expected this to continue in college (Camarena and Sarigiani, 2009; Cribb et al., 2019).

Families transitioning to postsecondary education need to adapt to changing educational services, student responsibilities and requirements, and legal protections; however, limited research exists on the first-hand experiences of students and their parents in this process. One study that focused on parent perspectives indicated that parents reported struggling to find a balance regarding how to best support their child’s independent living skills; while they wanted their children to be successful on their own, parents often stepped in and problem-solved when their child required assistance (Pena and Kocur, 2013). Parents are often responsible for supporting important skills and behaviors such as developing activities of daily living such as cooking and laundry (Hewitt, 2011; Cheak-Zamora et al., 2015). Encouraging and enforcing independence for their child can be difficult, but parents find it to be necessary (Pena and Kocur, 2013).

Moreover, parents must understand the differences in rights and expectations between high school and college, specifically the expectation of student independence (Morrison et al., 2009; Pena and Kocur, 2013). For instance, when students exit high school in the United States, they are no longer supported under IDEA and instead receive protections through the Subpart E of Section 504 of the Rehabilitation Act of 1973 and the Americans with Disabilities Act (ADA). Upon entering college, students must be able to self-advocate and self-disclose their disability in order to receive reasonable accommodations. While college students with disabilities work independently with disability service professionals to coordinate this process, parents may assist their students in various ways, including preparing for meetings or obtaining documentation of disability (Pena and Kocur, 2013; White et al., 2017).

## Study purpose

As noted, little is known about the perceptions of parents of children with 2e-ASD in regard to their student’s high school experiences and transition to college (Neumeister et al., 2013; Dare and Nowicki, 2015; Rubenstein et al., 2015) and the significant role that parents play in the secondary transition process, and the dearth of literature in this area supports the need to learn their perceptions of the success and challenges these families face. This information can be used by secondary personnel to improve the planning and delivery of the educational and transitional programs for students with 2e-ASD. It can also help teams to be more aware of non-academic factors that both enhance and interfere with education and transition of students with 2e-ASD. Therefore, the purpose of this study was to explore parents’ perspectives of their child’s best and most challenging experiences in high school, transition preparation, and suggestions about regarding how to improve college transition supports and services for students with 2e-ASD in future. The parents’ views about their child’s college experiences are described elsewhere (Madaus J. et al., 2022).

## Methods

Reis et al. (2021) conducted interviews with 40 college students with ASD, all of whom were enrolled in colleges or universities that are ranked among the top national institutions by U.S. News and World Report (2021). A broad approach to defining giftedness was utilized as students’ difficulties often mask their strengths (Reis et al., 2014). The students in that study had to indicate that they were (1) formally identified as gifted or talented in K-12 education, (2) participated in gifted education programs or services, or (3) participated in accelerated and advanced classes in high school. They also had to indicate that they had been diagnosed with ASD. Each student had also disclosed their disability to their college disability services offices and was receiving a range of services and accommodations based on their perceived needs at the time of the interview. For example, 19 of the 40 students reported using extended test time (Madaus J. W. et al., 2022). The students also had a range of semester standing at the time of the interviews, with 23% being first year students, 13% being second year students, 20% being third year students, 15% being fourth year students, and 10% being recent graduates. In total, 27 of the students were identified as male, nine identified as female, and four identified as non-binary or transgender. More details about the student demographics can be found in Reis et al. (2021).

After the interviews were completed, the students were emailed follow-up questions related to the study, and they were also asked if they would give permission for the researchers to reach out to their parents or guardians to conduct interviews about their parents’ perceptions of the students’ transition to college and college experiences (this request, and all procedures that are described later were approved by the Institutional Review Board at the institution where the research was conducted). This purposive sampling was used in order to allow comparisons between the student and parent perceptions. A total of 15 students gave permission and provided their parents’ email address or phone number, and nine of those parents agreed to participate in the present study. An additional parent who learned about the study at a professional conference volunteered to participate. This parent’s child was also attending a university that was ranked in the top 50 of regional universities by U.S. News and World Report (2021), and during the course of the interview, the parent disclosed that his son was tested in the very superior range on an IQ test when he was in elementary school.

## Study procedures

A semi-structured interview protocol was developed that paralleled the protocol used by Madaus J. W. et al. (2022). The parents were emailed a copy of the interview questions to enable them to reflect on them prior to the interview. The first author conducted nine of the interviews by phone and one *via* videoconference at the parent’s request. Interviews took place between August and November 2021 and, on average, lasted 46 min (SD 14.3 min). Each interview was recorded and transcribed. The transcriptions were reviewed for accuracy and checked against the recorded interviews. The authors decided after coding and analyzing these interviews that data saturation had been reached as they continued to “hear and see the same things over and over again,” and “no new information surface(d)” (Merriam and Tisdell, 2016) as data were analyzed.

Transcriptions were provided to each author, who then performed a thematic analysis, a process that identifies, analyzes, and describes themes found within the data (Braun and Clarke, 2006). First, each author independently read through the transcripts and documented reflective memos, which included initial reactions to the data and potential codes. Next, each assigned initial codes, often based on participants’ own words, to key aspects of the transcripts that related to the research questions. The authors subsequently shared initial codes with each other and came to agreement on any coding discrepancies. Codes were next grouped according to similarities and organized into tentative themes individually by each author, before they met to review findings, resolve any discrepancies, and refine themes. The themes were also cross-referenced with the individual codes contained in each to ensure the themes accurately encompassed the data.

**FIGURE 1:**
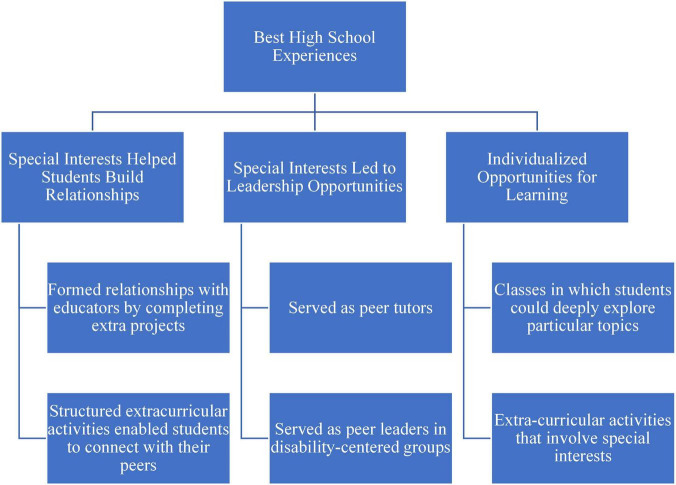
Coding map of the best high school experiences.

**FIGURE 2:**
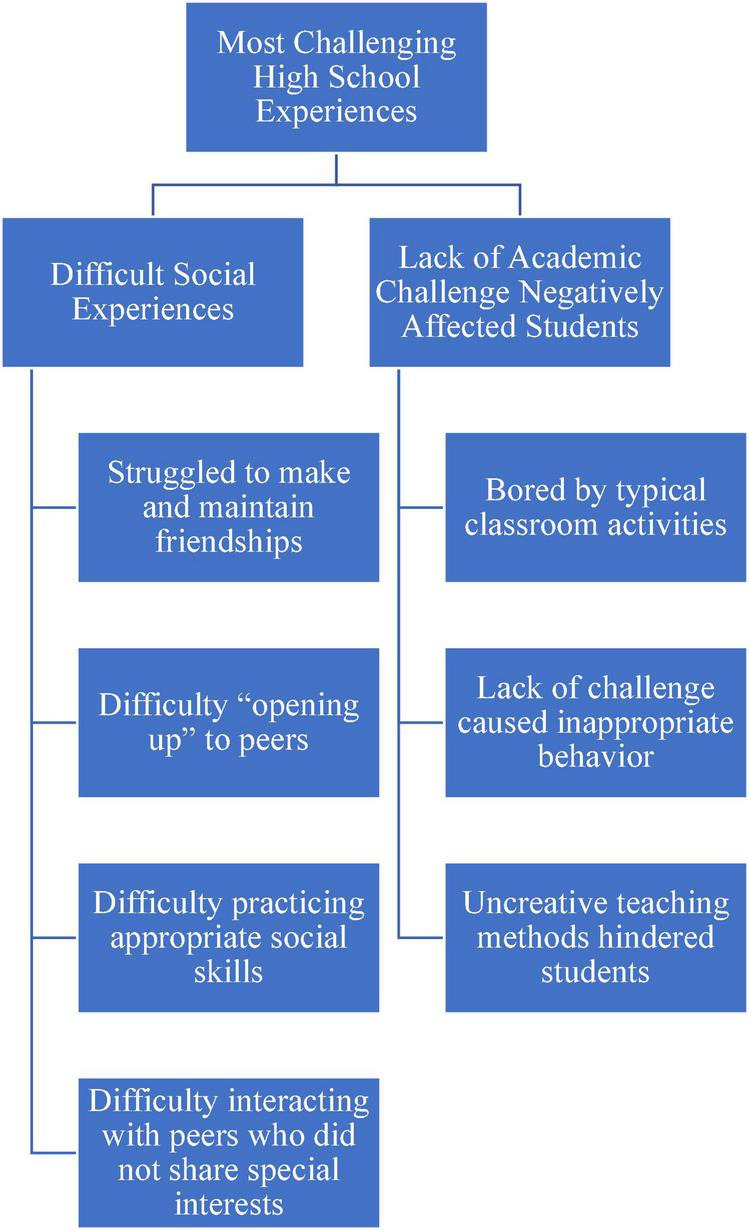
Coding map of the most challenging high school experiences.

**FIGURE 3:**
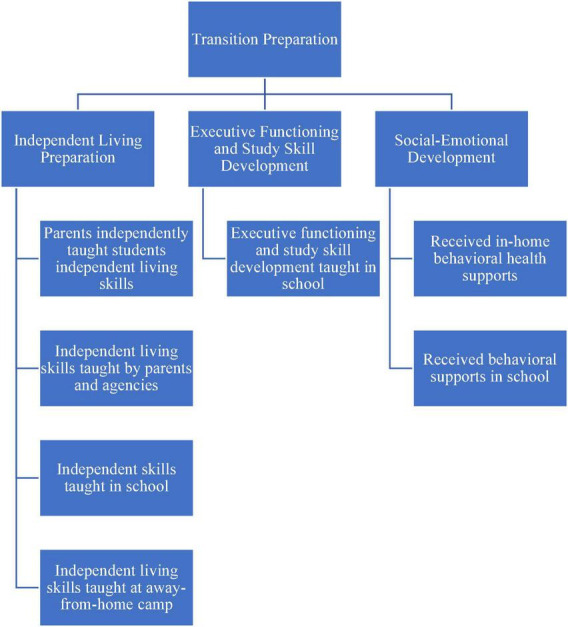
Transition preparation methods described.

**FIGURE 4:**
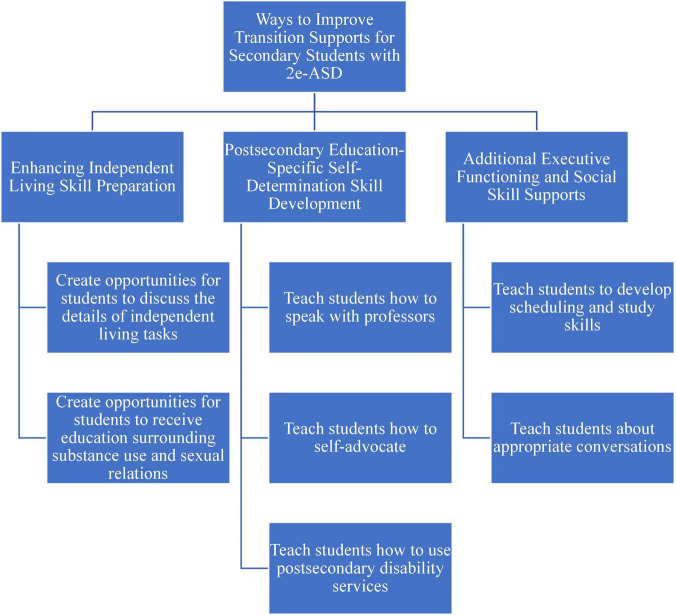
Suggestions for improving transition supports.

The themes and the corresponding codes were shared with each of the participating parents, and they were offered the opportunity to make any corrections or to provide feedback. None offered corrections or disputed the themes. A visual map of the themes and corresponding codes can be found in Figures 1–4.

## Sample

A total of 10 parents were interviewed as part of the study, six of whom were female and four of whom were male. In total, eight of the students discussed identified with the pronouns he/him, one identified with she/her, and one with the pronoun they. All 10 parents interviewed confirmed that their child experiences ASD and were asked the age or time in school at which their children received an initial ASD diagnosis. This occurred in early childhood (14 months) for one student, in elementary school for two students (ages 5 and 8 years), in late middle school for one, and in high school for two. Four parents did not provide the exact age at which their children were diagnosed, but instead gave a range of grades or years. Six parents also shared that their children received a gifted diagnosis but did not specify at what age. The remaining four parents said that their children were not diagnosed as gifted, but among them, three parents’ children took advanced placement courses and the other took honors courses in high school. In addition, the parents discussed co-occurring disabilities (the terms listed were used by the participants) that their children experienced, including anxiety disorders (mentioned by four parents), depression (mentioned by two), and the following disorders, each of which were only mentioned by one parent: sensory processing disorder, oppositional defiant disorder, obsessive compulsive disorder, mood disorder, and learning disability. Also, four parents mentioned that their children did not experience any co-occurring disabilities. A total of five students attended public high schools, and three others attended private high schools that focus on neurodiverse learners; two students attended a combination of these types of schools.

## Credibility measures

To ensure trustworthiness of findings, several credibility measures were used. The researchers performed researcher triangulation, or continually reflected on their own as well as each other’s analyses at every step of the research process to form consensus on codes and themes and reduce bias in analysis. In addition, the research methods used are richly described and accompanied by a coding tree that demonstrates the authors’ analytical process, enabling readers to understand how conclusions were reached. The authors also included rich and thick descriptions of participants’ responses, including quotations, to “provide evidence for researchers’ interpretations and conclusions” (Brantlinger et al., 2005, p. 201).

## Language

A debate currently exists regarding the preferred language used to describe people with autism/autistic people. Some individuals use person-first language to describe themselves, placing the “person” before the autism label (e.g., person with autism). By contrast, many autistic individuals prefer to describe themselves using identity-first language, which positions “autistic” before the person (e.g., autistic person; Vivanti, 2020). The parents who were interviewed for this article primarily referred to their children using person-first language, and as such, similar language will be applied throughout this article.

## Results

Themes from the analysis related to parent responses regarding their child’s best and most challenging aspects of high school are presented. In addition, we provide an analysis of the parents’ discussion of the transition preparation their child received and their suggestions for improving transition supports for students with 2e-ASD.

## Best high school experiences

Parents described their perceptions of their child’s best high school experiences, which included opportunities for individualized learning, and relationships and leadership roles that grew from these experiences (Figure 1).

### Individualized opportunities for learning

The first theme, mentioned by eight parents, was that one of the most positive aspects of high school was when their child received individualized opportunities for learning. Regardless of the setting (public or private schools) in which the students learned, the parents described their students appreciating opportunities to individualize their education, especially when that meant incorporating their special interests. The parents described their students flourishing when they took classes in which they could deeply explore particular topics or when they participated in extracurricular activities that involved their special interests. One parent said, “it’s like he did great in certain projects. He did great in places where he could, sort of you know, really throw himself into it.” As this student’s specialty interest or skill involved acting, the parent went on to describe how the student completed school projects by learning about the history of famous individuals and performing as that person. Another parent shared how a teacher created opportunities for her son to share his specialized knowledge with the class. He explained, “the teacher had him go up there and explain everything to him. He knows so much about the topic or whatever, the teacher will have him to go up there in front of class … he always loved it.”

### Special interests helped students build relationships

Studying or exploring special interests were also a source of relationship building for the students. Several parents expressed how their children formed relationships with educators by completing extra projects or sharing hobbies. One said,

My child academically, there are certain times they’ve studied a topic and written copiously that, they did a, such a substantial, mature level that they were elated. They felt like they had true intellectual rapport with the teacher. It was just a total synthesis. Seeing any time there was a sort of total connection, a powerful academic connection was gratifying for the teacher, for me, and I was so happy for my child because they had so much unmet potential.

Participating in structured extracurricular activities also enabled the students to connect with their peers with similar interests. The parents indicated that their students attended activities relating to theatre or music (4), sports (4), service activities (2), or special interests, including chess, history, and robotics, and tended to form small social groups with other students who were also participating. One parent said,

For high school he found his group of friends and he was happy with them and they did activities … they did gaming and they watched movies and talked about movies. And some of them were in the drama club, some of them weren’t and he, but he was just happy with it just being with his small group of friends that all have the same interests.

### Special interests led to leadership opportunities

A total of five parents described how their students’ advanced understanding of certain subjects or connections to peer groups resulted in leadership opportunities. A parent described that her son tutored students without disabilities. Another parent shared how her son’s peers perceived him as a leader. She said of her son at his high school,

He was really, he was a role model there. He had … a lot of friends and people really looked up to him and respected him. So I think … he built his self-esteem, which really made a big difference.

Also, three other parents shared that their students participated in groups or activities for students with disabilities, including Best Buddies, where their students served as peer leaders. A parent said of her son’s involvement with Best Buddies:

He certainly was able to kind of step in and then not just be someone who was kind of being attended to, but also sort of helping out. He took a leadership role in there as well. So him getting to be an events coordinator or something like that, you know, it with people who knew him and kind of expected that.

## Most challenging high school experiences

The parents shared their perceptions of their children’s most challenging high school experiences, which involved social interaction and not being challenged by academic work (Figure 2).

### Difficult social experiences

A total of seven parents recounted those social experiences encompassed the most challenging aspect of high school for their students. The parents described their students struggling to make and maintain friendships. One parent shared,

It was when she was in a neurotypical school and I could see the kids were kind of ostracizing her and then I would sign her up for girl scouts or other things and they would never gravitate toward her.

While one parent discussed her student was able to establish “one-on-one friendships,” she also noted that friendships were harder to maintain, stating “(the student) always wanted to do more things with them (friends) but they would pull away … it was a one-way friendship so it just sort of faded out.” The parents also related that their students had difficulty “opening up” to peers, practicing appropriate social skills, and especially interacting with peers who did not share their special interests. One parent said,

Like if you’re not on it, what he’s trying to do, he’s not gonna talk to you. Like he’s a history buff, he loves animals, so he’s like an academic guy. If you’re not, he’s not gonna pay you no mind.

### Lack of academic challenge negatively affected students

The next most commonly discussed challenge experienced by students in high school included not being challenged by academic work. A total of four parents described that their students were “bored” by typical classroom activities. A parent shared that the school could have “pushed (her son) a little harder” to keep him engaged, and another described that a lack of challenge caused her daughter to behave inappropriately, hiding under a desk. Referencing standard methods of teaching, including stationary learning, two parents indicated how these “uncreative teaching methods” hindered their students’ education. This approach to instruction inspired one student to transition out of his school to a private school for neurodiverse learners. His parent described his struggle:

He just can’t sit for 45 min and listen and then, you know, and then just sit and do their kind of rote homework. And especially because it takes him so much longer, you know, he had all the concessions for the testing, but it started to get really, really challenging having him in public school.

Another parent concurred and indicated that high school teachers “didn’t get” her son’s learning needs, which resulted in her son being “greatly robbed of accessing a proper education.”

## Transition preparation

The parents recounted that their students’ transition preparation revolved around four main areas: independent living skills, executive function and study skills, and social emotional development (Figure 3).

### Independent living preparation

The parents reported that home was the main setting in which students learned and practiced independent living skills. A total of two parents discussed how they worked independently with their children to help them learn key independent living skills, such as cooking, doing laundry, and creating and maintaining a budget. However, two other parents indicated that while their students received independent living skills training at home, the training was provided by an agency. Overall, the parents expressed the importance of their students having opportunities to practice independent living skills and “being responsible” while still in the safe settings of their homes. Describing services provided by her son’s high school, one mother explained that while her teenager’s high school offered independent living skills supports, only some of it stayed with him for long term. Another parent stated that her child attended day camps to hone life skills before transitioning to postsecondary education. Also, two other parents described day or residential camps that their students attended; however, these camps were focused on special interests, such as husbandry or film, and did not necessarily intended to foster independent living skills. In addition, two parents shared that their children attended camps that specifically aimed to foster transition or social-emotional skills. A student participated in a camp that helped students develop executive functioning abilities, including time management and organizational skills.

### Executive functioning and study skill development

Several parents discussed challenges that their children experienced related to executive functioning. As executive functioning abilities influence a range of academic areas, the parents described that their children’s executive functioning deficits presented multiple challenges. Commonly mentioned executive functioning deficits included regulating emotions, such as responding to conflicts, impulsivity, time management and organization, and most often, turning in assignments. A parent described that his son “does everything and does the work and he tells you exactly what he did. He just doesn’t know where he put it.”

Therefore, executive functioning and study skill development were another focal points of students’ transition preparation. While executive functioning skills, including planning and time management, could be practiced in conjunction with independent living skills, study skills development mainly occurred in high school classrooms. Primary areas of skill development the parents described included selecting effective learning strategies, such as note taking, developing and sticking to schedules, and organizational strategies. A parent noted how specific learning skill development was crucial for her student to develop effective strategies and stop practicing those that are not beneficial. She said, “so it’s choosing habits that will be most beneficial and sort of identifying when they’re not going particularly well, or efficiently that he can, sometimes he’s just stuck doing something cause he’s done that in the past.”

### Social-emotional development

The final area of transition preparation commonly mentioned by the parents included social-emotional development. A total of five parents indicated that as their children struggled with social skills or emotional regulation, they each received additional support in these areas. A student worked with an in-home behavioral health supports, or therapy, to help him develop social skills as well as coping mechanisms; three other students received similar behavioral supports in their high schools, including those addressing how to effectively deal with anger and frustration, but especially how to manage social situations. A parent described a high school social skills group in which her son participated. She said, “in the social skills group, he learned how to meet new people that you haven’t met before and how to have conversations.” The parent went on to explain how these skills were useful preparation for college.

## Ways to improve transition supports for secondary students with 2e-ASD

The parents suggested bolstering three key areas of transition supports for students with 2e-ASD, including those that help students develop independent living, self-determination, and enhanced work on executive functioning and social skills (Figure 4).

### Enhancing independent living skill preparation

The parents explained that the students would benefit from not only learning basic independent living skills but also having opportunities to discuss the details of these tasks. A parent provided the following examples of teaching the “specifics” of tasks:

You can’t put a metal object in a microwave … or laundry, water temperature setting. You don’t want all your underwear to turn pink, you gotta make it cold. So like maybe half a day of teaching them how to … use the washing machine and dryer, making the bed.

In addition to learning typical independent living skills, two parents emphasized that the students also need to receive education surrounding substance use and sexual relations. They described how the students would be inevitably exposed to the both during college and, therefore, would benefit from preemptive knowledge about each. A parent described how students on the spectrum may have difficulty interpreting social cues regarding sexuality. She said,

Like if your roommate says, “Hey, can I have couple hours to myself with my girlfriend?” I feel like a lot of kids on the spectrum are, not socially advanced as kids who are not on the spectrum … but, you know, college is … you’ve got a bunch of 18, 19, 20 year olds, and I think kids on the spectrum need to know this aspect of the social skills.

These two parents also advised helping students on the spectrum understand how to react if they see peers using substance. One of them proposed,

What are you supposed to do when you see somebody smoking pot, cigarettes, drinking, all that stuff, that stuff that comes with college? There’s gotta be a sensible way to teach kids on the spectrum, how to deal with situations like that.

### Postsecondary education-specific self-determination skill development

The parents related self-determination skills to the need to communicate with professors, advisors, and other postsecondary professionals, and how to identify needs and locate resources to fulfill them, especially those offered by disability services. First, the parents shared that the students need to know not only how to speak with professors but also how to do so at appropriate times, such as office hours. A parent suggested that students receive instruction regarding:

How to talk to professors, how to you know, utilize the office hours. You can’t only when you’re panicking! Just because you’re panicking, you can’t just call or, you know, show up that the professor’s office. There are time slots that you can do that.

Another parent spoke about the need for his child to self-advocate when he needed a break. He said that his student needed to be able to say,

I need to stop now. And there’s a good reason for it. I’ll be back in a minute, but I got to compose myself. Please understand that, you know, I’m not appearing unprofessional right now on purpose. This is something that, again, I have a disability.

The parents also discussed the need for students to know what disability services offices can offer, as well as understand the protocols and procedures to obtain support.

### Additional executive functioning and social skill supports

Additional two additional skills the parents believed were critical for their students to develop before transitioning to college were executive functioning and social skills supports. Specifically, learning to create and maintain a schedule, and develop and refine study skills that meet the student’s needs were mentioned. Regarding social skills, the parents mentioned teaching students not only how to meet and talk to new people but also for them to learn about how the appropriateness of conversation topics depends on the setting. A parent noted that her son was asked to leave a group on campus because he would make “politically incorrect jokes” and that learning to identify such situations was a skill he continues to develop. She described, “so he belonged in the gaming club at (institution) but he was kicked out from the club because one of the problems (name) has is that he would, he would say politically incorrect jokes. And I don’t think that was appreciated.” Another parent provided the example of not discussing politics when learning how to complete job tasks in a work training setting. She said,

they had to tell her you can’t talk about politics. It was like not a common thing. And so that might be something that could help with kids when they go from high school to college and talking about jobs and what’s appropriate to talk about or not.

## Discussion

The parents shared their perceptions of their students’ most positive and challenging high school experiences. They also reflected on the transition preparation their children received and ways these services could be improved. The parents reported that the students’ best experiences involved individualized opportunities for learning, namely, those that enabled the students to explore areas of special interest or build relationships with others who also engaged with these topics. By contrast, the parents identified poor social experiences and a lack of academic challenge in high school as areas of difficulty for their students. These observations match the findings of Reis et al. (2021), who interviewed 40 academically talented college students with ASD (and who as noted previously served as a conduit for the development of the current sample). Ensuring opportunities for academically talented students with ASD to participate in individualized learning experiences in high school has the potential to minimize both areas of challenge. Through individualized learning, the students can explore subjects deeply and can engage with others with similar interests. There is also a need to prioritize opportunities for individualized learning in transition planning for postsecondary education and, as noted, for student IEPs to focus more on the strengths and interests of students (Reis et al., 2014).

The parents conveyed that their students’ transition preparation focused on developing independent living, executive functioning, and social skills. While they noted that their students progressed to varying degrees in these areas, they also suggested ways transition services could be enhanced. Recommendations included discussing the nuances of independent living and social skills, as opposed to only general skill development, exposing students to information regarding substance use and sexual education, and developing self-determination skills, specifically those involving how to communicate with professors and advisors, how to use available disability services, and how to practice self-advocacy and college-specific executive functioning skills, such as creating and maintaining schedules. The parents also pointed to the importance of the students being able to frequently practice these skills while in high school, as well as the importance of exposing the students to independent living skills in a controlled way, before moving to college.

## Implications for professionals involved in transition planning

Based on the feedback from the parents of academically talented college students with ASD, this section presents suggestions for transition professionals, including educators, vocational rehabilitation counselors, and other related professionals.

### Facilitate individualized postsecondary experiences

As these students with 2e-ASD thrived when provided with individualized learning experiences in high school, similar opportunities should be incorporated into transition planning and identified in the postsecondary settings the students plan to attend. Research has found that college students with ASD appreciate that they can pursue classes related to their areas of interests and potentially adopt majors in these fields (Madaus J. et al., 2022). Transition professionals can help the students to identify colleges with majors of interests by encouraging them to complete interest inventories and explore different programs offered by colleges online. Once a list of colleges is identified, transition professionals can encourage the students to research related student organizations and clubs, as well as some research or service projects currently carried out at the institutions and learn more about how the students can become involved. The students could be encouraged to reach out to club or organization leaders. These suggestions can help transitioning students to find ways to expand their learning outside of the classroom, as well as facilitate relationships with others who share their interests.

### Help students develop and practice social skills

The parents recognized social and executive functioning skills as common areas of challenge for their children. Difficulties in these areas can inhibit students from participating in activities that were identified as beneficial for students with 2e-ASD, including individualized learning experiences and structured social activities. As noted, transition professionals can help guide the students to learn more about academic and non-academic opportunities available at colleges, but they can also assist students in developing skills in high school that will help foster these connections in college. The students could be exposed to the range of clubs and student organizations within their school and community and be encouraged to become involved in ones that are of high interest. Transition professionals could help them navigate the hidden and “soft skills” needed to successfully interact socially. For example, if a club meeting typically begins with an ice breaker, presents an agenda, and performs a group activity, the professionals can discuss what appropriate social skills look like for each stage of the meeting and allow students to practices these skills.

### Help students develop and practice executive functioning skills

Another important aspect of transition planning for postsecondary education can involve helping students to learn and continually practice executive functioning skills. The parents noted that their students would have benefited from improved skills in time management, organization, and both work competition and work submission. These are all practical skills that can be embedded into a student’s IEP, and into a secondary program of study, if a student is not on an IEP. Transition professionals can also help students reflect on what types of supports they might benefit from in college and guide students to contact campus disability services offices to determine if supports are available to develop executive functioning skills, such as meeting with disability service professionals or peer mentors. Some colleges, such as the University of Maine, also offer online social skills training that enable students to practice social skills using virtual role-plays and behavioral exercises (University of Maine College of Education and Human Development, 2021), and transition professionals could work to connect students with such resources.

### Develop awareness of available disability services in college

College disability services offices provide a range of services and reasonable accommodations that typically apply to class work or assessments, such as extra time or a reduced distraction environment for exams, notetakers, or residential needs, such as a single dormitory (Gelbar et al., 2015). Some may offer more intensive small group or one-to-one support with disability service specialists, but this may come with an additional fee (Brown et al., 2012). Transition professionals can work with students to reflect on their current accommodation use and how each helps currently. With this baseline awareness, the students could then reflect on what types of accommodations might be useful in college and what degree of support they might need. If students require coaching services, particularly to support executive functioning skills as discussed previously, it is important for them to inquire if these supports will be available through disability services and/or if there is an extra fee to access them. If they are not available, transition professionals should work with students to find other ways to support this need. They can investigate with students if there are peer supports, such as peer tutors, who can assist students with planning and study skills, if hiring a private executive functioning coach would be feasible for the student, or if assistive technology, such as apps that support organization, planning, prioritizing, or remembering deadlines, would be helpful (Gelbar et al., 2015). The parents also talked about the importance of their children being able to talk to their college faculty about their learning needs and particular circumstances. In order to practice this, secondary students should be encouraged and supported to talk to their teachers regarding their accommodation needs and to be part of the process to set these up and evaluate their effectiveness.

Another potential support for twice exceptional students with ASD includes connecting with agencies that offer skill development and community for adults with autism, such as the Autism/Asperger’s Network (AANE). AANE, for example, offers support groups, interview preparation, life coaching, and social events, all of which can support the social development of adults with ASD. In addition, coaching students to research these opportunities as independently as possible can also allow them to practice the self-advocacy skills they will need in college.

## Limitations

As with most qualitative research, this study contained small sample size, which limits its generalizability. The participants may also have felt the need to offer socially desirable responses, potentially influencing the information they shared with researchers. In addition, there was potential for researcher bias to influence the interpretation of study results. The researchers attempted to minimize potential bias by individually coding the data before comparing results to determine themes. We did not collect information about the parent participants’ demographics beyond gender (e.g., race/ethnicity, socioeconomic status, and level of education), which presents some potential limitations to the transferability of the results.

## Summary

The perceptions of the parents of academically talented college students with ASD related to the college transition process provide valuable information for both secondary school personnel and the families of younger students with the potential to attend college. Focused early transition supports and opportunities related to instruction, extracurricular enrichment activities, and practicing executive functioning and independent living skills may enhance successful transition to college for this growing cohort of students.

## Data availability statement

The raw data supporting the conclusions of this article will be made available by the authors, without undue reservation.

## Ethics statement

The studies involving human participants were reviewed and approved by the University of Connecticut Institutional Review Board. The ethics committee waived the requirement of written informed consent for participation.

## Author contributions

JM conducted the interviews, participated in the data analysis and in all stages of the manuscript development. ET and SL participated the data analysis and wrote the analysis and results section. NG assisted the development of the interview protocol and all parts of the manuscript development. All authors contributed to the article and approved the submitted version.

## Footnotes

1 The participants in this study tended to use person-first language. Therefore, person-first language will be used throughout.
